# Making community voices heard in a research–health service alliance, the evolving role of the Community Advisory Group: a case study from the members’ perspective

**DOI:** 10.1186/s40900-021-00326-6

**Published:** 2021-11-27

**Authors:** Janet L. Wale, Louisa Di Pietro, Heather Renton, Margaret Sahhar, Christine Walker, Pamela Williams, Karen Meehan, Elly Lynch, Melissa Martyn, Jane Bell, Ingrid Winship, Clara L. Gaff

**Affiliations:** 1 Brunswick, Australia; 2 Genetic Support Network of Victoria, 50 Flemington Road, Parkville, VIC 3052 Australia; 3 Syndromes Without A Name (SWAN) – Australia, PO Box 390, Fairfield, VIC 3078 Australia; 4 Malvern, Australia; 5 Chronic Illness Alliance, Melbourne, Australia; 6 Mount Waverley, Australia; 7 Melbourne Genomics Health Alliance, 1G Royal Parade, Parkville, VIC 3052 Australia; 8 Department of Paediatrics (Royal Children’s Hospital), Faculty of Medicine Dentistry and Health Sciences, The University of Melbourne, Parkville, Australia; 9 Melbourne, Australia; 10 The Royal Melbourne Hospital, Grattan Street, Parkville, VIC 3052 Australia

**Keywords:** Community advisory group, Community involvement, Research-to-clinical study, Genomics, Service implementation

## Abstract

**Background:**

The Melbourne Genomics Health Alliance (the Alliance) is a collaboration of leading hospitals, research and academic organisations, supported by its member organisations and the Victorian Government. The Alliance was set up by its members in 2013 to steer the translation of genomics, making it an integral part of health care in Victoria, Australia. The Community Advisory Group (CAG) was formed soon after, to give input and advice across the program. This was to ensure consideration of community values, perspectives and priorities, and knowledge translation for patient care. The CAG was charged with providing a strong community voice for the duration of the program. Appointed members were experienced consumer advocates with developed connections to the community.

**Main body:**

The Alliance progressed from an initial Demonstration Project (2013–2015) to a multifaceted program (2016–2020). The CAG worked strategically to help address complex issues, for example, communication, privacy, informed consent, ethics, patient experience, measurement and evaluation standards and policies, data storage and re-use of genomic data. Many aspects of translating genomics into routine care have been tackled, such as communicating with patients invited to have genomic testing, or their caregivers, and obtaining informed consent, clinical questions across 16 areas of health care, training and education of health and laboratory professionals, genomic data management and data-sharing. Evidence generated around clinical utility and cost-effectiveness led to government funding of testing for complex genetic conditions in children.

**Conclusion:**

The CAG activities, recorded in a CAG-inspired Activity register, span the full spectrum of information sharing and consultation to co-design and partnership. The CAG were involved at multiple levels of participation and in all tiers of activity including governance, development of policies and procedures, program planning and evaluation. Working relationships were built up and a level of trust instilled to advance the Alliance work program in ensuring an effective patient-care model of delivery of genomics. CAG input into project deliverables has been tangible. Less tangible contributions included presentations at external meetings and conferences, direct interactions at meetings with Alliance members, interactions with visitors and external experts, taking part in consultations with experts, state and federal government.

## Plain English summary

Melbourne Genomics Health Alliance was established in 2013 to steer genomics into health care in Victoria, Australia. The Community Advisory Group (CAG) was formed soon after to provide advice and insights from the patient perspective. The CAG has added value to the Alliance’s complex research-to-clinical service program of work over eight years to date. Following an explanation of the program, the CAG members identified priority areas and mechanisms for their involvement. Areas that members were involved in included: communication, visual identity and website, patient portal and its evaluation, information management, consent processes, laboratory requirements, tools for patient experience and quality of life measures, predictive health issues study, storage and sharing of data, databases, CAG Communication Plan, the Patient Guide, role with Victorian Government Department of Health and Human Services, implementation plan, workshop to upskill patient advocates, financial and strategic planning. Members also presented on the role of the CAG at conferences and symposia. The balanced, trusting relationship that developed between the CAG, the Program Team and its governance structure was of great value to and an achievement for the Alliance. CAG input into project deliverables and impact was recorded in a CAG inspired Activity Register and has been very tangible. Their less tangible contribution to the project is also important. Contributions included presentations at external meetings, direct interactions at annual meetings with Alliance members, interactions with visitors and external experts, taking part in consultations with experts, state and federal government. These provided opportunities to influence mindsets.

## Background

Genetics and genomics are rapidly developing highly technical areas of medicine including the study of a person’s genes and the technology needed for analysis and interpretation. Genomics raises complex ethical issues that require public involvement by individuals, families and communities from diverse populations to fully understand the ethical, social and economic implications [1]. The introduction of genomics into clinical medicine also requires clinicians to upskill to be both able to adequately inform their clinical care decisions and provide explanations of the risks and challenges of genetic information to their patients. Value can then be created when the quality of the health care and subsequent health outcomes are improved [1]. Patient and public involvement and engagement in health service delivery and research has become widely accepted in many countries such that the impact and contributions of different models of engagement can be compared [1]. The technical nature of the field means that this is a challenging area for meaningful involvement. A review of activities has identified that involvement includes different people from trial participants through to committee members as individuals and representatives of patient groups, and contributors to public debates. Furthermore, current genomic research covers a spectrum of activities and for different purposes [2].

An analysis of key literature on effective public participation in ‘health policy and planning’ identified three issues. The diversity of aims and forms of involvement methodologies used makes it difficult when considering ‘what is effective’; the definition of success depends on whose perspective is being considered; and identifying the endpoint to measure ‘outcome’ [3]. As an example, the United Kingdom (UK) 100,000 Genomes Project set out to sequence the genomes of 100,000 National Health Service patients, to inform clinical practice; together with a research-focused goal to provide data for ‘scientific discovery’ [4]. Public support is required for the program to encourage recruitment of participants as well as to advance genomic research into clinical practice [5]. This project incorporates extensive patient and public involvement and education activities and has a National Participant panel that acts as an advisory body to the Genomics England Board to ensure that the health data available for research is being looked after and respectfully used in the best interests of the participants [6]. A current UK study that sets out to collect genomic data from 10,000 autistic people and their families has drawn major criticism for failing to consult with the autism community [7]. This demonstrates the essential elements of communication and accountability with patient and community input to ensure consideration of community values, perspectives and priorities, as well as the sensitivity to and power of large-scale genomic projects [7].

Engaging patients in their care can lead to enhanced service delivery and clinical governance, and inform patient and provider education and policies [8]. Meaningful engagement of health consumers and the development of an evidence-base on the roles they play can be used to inform best practices in research [9]. Reasons for communities to be engaged in genomics policy and service delivery development include to identify areas of agreement and disagreement and to gain an understanding of underlying factors including broader community values and aligning practice recommendations with societal needs and expectations [10]. Being engaged can increase overall genetic literacy as guidance is provided on implementation, transparency, and quality and trustworthiness of policies and service delivery programs [10]. At the same time, it is important to understand patients’ and communities’ experiences of engagement, both in research and in health service delivery [11].

In clinical care, genomics has the power to assist in diagnosing diseases that have been hitherto difficult to diagnose [12], with the subsequent determination of effective treatments. It also has the potential for an important role in reproductive planning [13]. The data generated from genome sequencing can be stored and re-analysed as our knowledge of associations with health conditions increases and new treatments become available. It therefore has the potential to inform research and health care [14]. An objective of the Alliance was to forge a path forward for patients, clinicians and researchers to benefit from the enormous potential of genomics [15]. The CAG was created by the Alliance leadership at the beginning of the program to work as part of the governance structure [16] for the purpose of gaining community trust.

In this case study we describe the CAG’s role in working with the Project Management Team on governance, design of programs, making incremental improvements and problem solving, as well as in communicating the work of the Alliance (Table 1). In this program the genomic testing was performed as part of clinical testing for patients within the Alliance member hospitals. The Alliance is a service provider–research initiative to introduce genomics into clinical practice for public hospitals within the state of Victoria in Australia. Patients were asked to consent to being part of the research program to provide genomic testing as part of their clinical care, where the testing could potentially better inform and enhance their care. Each of the Alliance member hospitals have a consumer advisory committee that reports to the hospital board in line with the state of Victoria Partnering in Healthcare framework [17]. Hospitals in Australia are accredited against National Safety and Quality Standards one of which is ‘Partnering with Consumers’ [18]. A CAG was formed as part of the Alliance right from the beginning of the program. We present the ways in which the CAG has added value and its evolving role to become a partnership-focussed model of engagement [19].

**Table 1:** Clinical areas covered by the flagships in the Melbourne Genomics Alliance program, in infants, children and adults

The five clinical areas included in the Demonstration Project phase were:
Hereditary neuropathies, conditions of the peripheral nervous system
Focal epilepsy
Hereditary colorectal cancer
Genetic conditions of childhood, in infants and children (Childhood Syndromes)
Bone marrow transplants in acute myeloid leukaemia (AML)
The six clinical areas of the Flagships in phase one of Horizon One, during the period 2016 to 2018:
Immunology
Dilated cardiomyopathy
Congenital deafness
Complex care in children
Advanced non-Hodgkin lymphoma
Advanced solid cancers
The five areas of the Flagships for phase two of Horizon One, during the period 2017 to 2019:
Bone marrow failure
Controlling superbugs – resistant microorganisms
Complex neurological and neurodegenerative diseases
Genetic kidney disease
Perinatal autopsy
And ‘Additional findings’ project

## Main body

### About the Melbourne Genomics Health Alliance program of work (Table 1, Fig. 1)

In 2013, the Alliance was formed by two leading metropolitan hospitals together with research and academic organisations to inform and drive the widespread complex changes needed to bring genomic medicine to patients [20, 21]. The aim was to guide clinical management and improve health outcomes within a sustainable health system. The program is being run in three consecutive phases. In this paper we cover the first two phases, a demonstration phase (2014–2015) to demonstrate the value of genomic testing, and a broadened Horizon One (2016–2019). The Final Phase (2021–2024) further extends the work of the Alliance to hospitals across urban and regional Victoria [22].

#### Demonstration phase: effectiveness-implementation design

The rate of detection of mutations, the impact on patient care measured as the number of patients whose care changed in response to the test results and the nature of the change, and costs were determined in five different clinical areas (flagships), selected by consensus among the Alliance members [21]. Common policies, standards and procedures across hospitals were set up from selection of patients, genetic counselling and obtaining consent, through to return of sequencing results and associated changes in clinical management. A targeted analysis approach was used where known genes related to the patient’s condition (using predefined gene lists) were analysed to reduce the chances of additional findings not related to the current health care the patient was receiving. Workforce development was an important component, with experiential learning for diagnostic laboratory staff, clinical specialists, and genetic counsellors, raising ethical, legal and technical issues [21]. Management of patient, consumer, carer and family hopes and expectations, privacy and storage of genomic data as well as evaluation of patient experiences were priority areas for the program. It was important to understand the views and experiences of patients and families undergoing genomic sequencing, and to explore the impact of genomic sequencing on their lives. The participants received genetic counselling before and after genomic sequencing in addition to the usual clinical investigations.

#### Horizon one (Fig. 1)

Three additional metropolitan hospitals joined the Alliance with a call to members for new collaborative clinical projects. A competitive process was set up to instil greater trust in the process and motivation by the participating clinicians [21]. Projects were short-listed by external reviewers based on the criteria of clinical utility, potential cost effectiveness, feasibility and collaboration. A short-term committee that included a member of the CAG selected the final flagships to ensure there was breadth across disease areas and member hospitals. The Alliance continued to use a common approach to delivering services, harnessing the latest research, building health workers’ skills and knowledge and ensuring appropriate access to quality information [23]. Overall, people seemed more willing to reach agreement on decisions where the outcome—a policy, guideline or software—was subject to evaluation [21]. Evidence generated on benefits, clinical utility and cost-effectiveness led to Health Technology Assessment (HTA) approval to reimburse testing for complex genetic conditions in children. Across HTA systems, patient advocates have important roles in informing and supporting HTA processes to improve health outcomes for patients, by providing patient submissions and sitting on appraisal committees [24].

Genomic testing offers potential for developments in health care but also risks for the privacy and autonomy of individuals and their families [25, 26]. The resulting data can be analysed for reasons unrelated to the original reason for testing, including predictive information on future diseases [14]. Two projects focussed on pre-emptive “Additional Genomics Findings”, which are alterations in genes that are associated with medically actionable and serious conditions. One project offered parents of a child undergoing sequencing to detect the cause of bilateral hearing loss the opportunity to find out about other treatable and non-treatable conditions that occur in childhood [27]. The second offered adults whose diagnostic testing was complete the opportunity to have their data re-analysed for actionable conditions that occur in adulthood [14]. A member of the CAG was involved in this project.

### About the Community Advisory Group (CAG)

The CAG members are experienced consumer advocates and come from the Alliance members’ community advisory committees and relevant community groups such as the Genetic Support Network Victoria, Chronic Illness Alliance, and Syndromes Without A Name Australia (SWAN). They have extensive background knowledge in the technical nature of genetics and genomics, quality of health care, and in medical research.

#### How the CAG was selected

The Executive Director/Program Lead and the Steering Committee member and first Chair of the CAG interviewed suggested members before inviting them to be on the CAG. CAG members brought knowledge regarding lived experience of disease, had differing roles in health care and policy and in working with diverse communities. They were able to represent broad groups of patients in Victoria. The CAG came with the attitude, as experienced community leaders, of ‘let’s work constructively together’ to enable a quality health service. The Alliance leadership were committed to having a successful CAG. CAG members were encouraged to be pro-active, willing to participate, allocate time and resources, and consistently undertake public participation. Patagonia and Ward propose that with sufficient knowledge to enable capacity to act, developing a strong relationship between themselves and others, and working together for ‘a common good’, such a group can develop trust and mutual respect [3].

We developed our well-balanced relationships because of our previous experience base in working in community organisations and with government bodies where we learnt the skills required to best achieve mutual aims; the value placed on our contributions by Alliance staff; plus our inclusion as part of work plans and strategic planning. (CAG member)

#### CAG process

The CAG meets formally on a quarterly basis, and also contributed regularly through e-mail, attending working groups, special activities and events. From mid-2015, CAG members were paid a modest honorarium to acknowledge their input and commitment.

#### CAG roles and responsibilities

CAG roles and responsibilities were around governance (for example, strategic planning and reporting, patient information and support, informed consent, management of databases and sharing of data); design (website and patient information, forms, questionnaires, participant studies and evaluation); problem solving; and enabling communications (see Table 2).

The CAG was able to connect us to the diversity we needed except in one instance, a project with diverse communities on data sharing. In the end we interviewed genetic counsellors working within the program. (Program Management Team member)

**Table 2:** Key activity areas of Community Advisory Group

In what activity	How involved
Governance	Selection of Flagships (Horizon One); design of patient test report (Demonstration project); review of research
Consent processes	Participated in discussions on dynamic consent, design and content of clinical consent, Participant Information Sheet and Consent Form
Communication, visual identity and website	Provide contacts and access to patient networks for real stories that were the basis of media stories, provide speakers, helped with website design and provided examples, website content, feedback on brand identity
Patient portal and evaluation	Advised and reviewed patient surveys and return rates, portal content during development together with access and navigation, patient-facing materials and information (visual media, glossary, navigate your results section)
Information management	System planning, test tracking for patient-facing portal, incremental levels of information while test results pending
Laboratory requirements	Stressed right from start importance of use of accredited laboratories to provide genomic testing
Tools for patient experience and quality of life measures	Actively participated in discussions on tools, addressed cultural/language diversity; design, data collection and analysis of evaluation cycle
Pre-emptive additional findings study	One designated CAG member was active member of study [10]
Storage and sharing of data, databases	Actively participated in discussions, workshop
CAG Communication Plan, the Patient Guide	Co-design of materials
Sharing role with Victorian Government Department of Health and Human Services	Representative attended meetings as non-member
Implementation plan	Consultation on and input into plan [18]
Workshop to inform/upskill patient advocates	Co-design and participation in workshop
Financial and strategic planning	Lobbying state government, input into priorities for funding business case
Presentations on the role of the CAG at external meetings	Direct involvement of members
Direct interactions with Alliance members at annual meetings	Direct involvement of members
Interactions with visitors and external experts	Direct involvement of members
Participation in consultations with external experts	Direct involvement of members
Final two-day symposium	Direct involvement of members

#### Selecting areas of involvement by CAG members

**Fig. 1:**
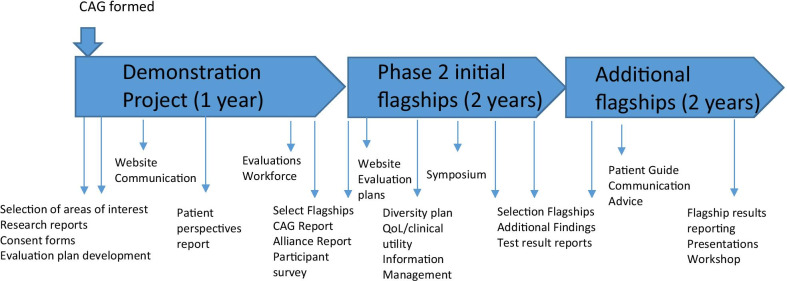
Community Advisory Group (CAG) activities throughout the work program of the Melbourne Genomics Health Alliance

At the first meeting the projects being undertaken by the Alliance were presented to the CAG for it to identify and prioritise what the CAG should and could be involved in. From there the mechanisms of engagement ranged from broad consultation to representation on working groups and participation in workshops. Participation as a group or individually was guided by the particular skills and knowledge of each of the CAG members. Community involvement is an iterative process that may not always be easy and needs time to grow. The roles of the CAG and its members varied in line with the Alliance program (see Fig. 1). Involvement covered the spectrum of levels from consultation through to co-design and partnership [28]. As the program developed, CAG members were kept informed of current activities by Program team members and received updates from flagships. In return they provided input and advice, took on aspects of the work for broader consultation and discussion, and endorsed actions, reports and documentation.

The CAG has been key to the development of patient-centred genomics information for the website.” “CAG member input has been instrumental in understanding the information needs of patients when they are undergoing genomic testing. The CAG has also significantly assisted in the development of online surveys that enable the Alliance to understand patient preferences for providing additional health information to supplement a genomic test. (Information Management, and Information and Communication Technology (ICT) Project Manager)

#### Leadership commitment

The Alliance demonstrated its commitment to the CAG by appointing an eminent research professor and practising clinical geneticist as the Chair for the first year. In addressing our questions related to genomics, research and clinical applications she assisted the CAG in making informed contributions to the work of the Alliance. CAG members also contributed to lectures and symposia, were active participants in working groups and advisory committees, and took part in policy discussions. They also had the opportunity to suggest national and international visitors. These activities ensured the members were informed. For Horizon One an independent chair who was experienced at a high level of governance and had worked previously with consumers was appointed. A similar process to that of identifying the CAG members was undertaken.

#### Activity register

The CAG set up a self-reported activity register to monitor its contributions to the outcomes of the program (see Table 2). In its first two years, CAG members (six people) completed timesheets and logged 250 h of contributions. The register was used to report to Alliance members, and in speaking about the role of the CAG, for example in poster presentations at the International Society for Quality in Health Care (ISQuA) 2017 International Conference and at the Consumers’ Health Forum (CHF) Summit on ‘Shifting Gears’, in March 2021 [29, 30]. The latter informed the present paper.

#### Self-appraisal of the work of the CAG

At completion of Horizon One, a questionnaire was developed by a CAG member together with members of the Project Management Team. The purpose of the questionnaire was to provide feedback on the function of the CAG to date and to consider what changes may be needed for going forward (see Table 3). One identified omission was in providing orientation to the most recent member of the CAG, who joined at the beginning of Horizon One and when the existing members had settled into their ‘roles and responsibilities’.

As a CAG, we have endeavoured to acquit ourselves to the best of our abilities according to individual skills, limitations around time, and availability—attending meetings, presentations and forums, responding to requests for experiential information through access to member networks, evaluating documents and public interfaces, and providing advice and assistance to the Project Team.” (CAG member)

We believe the input and advice we provided during Phase 1 was sound and well received, with many of the CAG’s recommendations having been taken on board and actioned. (CAG member)

**Table 3:** Identified mechanisms for promoting CAG involvement and partnership, and lessons learned

1. Careful selection of CAG members
A good mix of expertise, advocacy and lived experience
Firm commitment to working together—experienced in working in community organisations, and with government bodies where members learnt the skills required to achieve mutual aims
Health equity focused
The ability to give voice to consumer concerns; to communicate successfully with other professionals
CAG members empowered to set program of work, as a CAG and individually where special interests lay
2. Creating a receptive environment
Use of democratic dialogue
Independent chair who kept to time and structured meetings
Set opportunities for interaction – regular defined meeting schedule; by e-mail between meetings
CAG Chair and Project Management Team support
Carefully selected co-ordinator from Project Team
Consulted on approach to be taken before decisions made
Flexibility in the levels and approaches of involvement
Engaged in multiple ways, utilising individuals’ strengths
Well prepared and informative presentations from Project Team
Well-presented updates from Flagships
Given time and opportunity to develop strong and trusting relationships
Value seen to be placed on CAG contributions
Activities register to record activities, enable identification of outcomes of involvement
Built in reward mechanisms such honoraria, enabling workshop development, posters at conferences, presentations etc.
3. Leadership commitment
Commitment to and resources for CAG
Leadership attended and actively involved in meetings
CAG members attended and actively contributed to key Alliance external and visitor meetings and events
Regular updates on the Program and funding
*Limitations, challenges and lessons learned*
No dissenting voice present
No consideration of a more diverse membership including men, youth, members from culturally diverse backgrounds including Aboriginal and Torres Strait Islander people, and from rural and regional areas to provide additional aspects and points of view
The need to establish credibility and overcome scepticism from some professionals; that our credentials and comments are valid
Co-ordinators had different backgrounds (genetic counsellor, communications, then researcher) with unknown implications for the group
No induction to the CAG for new members
No mechanism to check impact and involvement across research activities
No formal evaluation undertaken of the CAG and its place in the Alliance

#### Additional activities

The CAG played a strong community advocacy role for the Alliance, for example in providing patient stories for use in public communications, speaking with politicians, and providing informed input on government documents (both state and federal) as well as in their own spheres of activity.

CAG’s support and input has been particularly important in helping convey the power of genomics to assist patients and their families. In this way, CAG has strengthened public communication of the Alliance’s work and the case for government funding. (Chair, Steering Committee, Demonstration phase)

Gaps in knowledge of local patient support groups about genomics was identified by CAG members as an area to address. The CAG worked with the Program Management Team to design and present a workshop aimed at upskilling patient advocates about genomics and related issues [31].

#### Impact

The CAG presented its input to the Alliance members during the Demonstration project [32].

The members of the CAG provided really practical and useful insights into so many aspects of the Alliance’s work. Their involvement in media and educational activities, testing of a range of patient tools and surveys and contributions to the overall project plan were particularly valuable. (Clinical Project Manager)

At the end of Horizon One, the feedback from the Program Leader and her team was that the CAG input across the program resulted in better research and health care:

The CAG influenced many of the decisions that were made and also the quality of the output in terms of ‘deliverables’. (Program Leader)

In the final questionnaire sent to CAG members, the Chair of the CAG stated:

*“I have an appreciation of the challenges and opportunities facing genomics in Victoria and Australia and the critical role that the CAG plays in bringing consumer perspectives to the organisation’s deliberations and decisions*. “

The CAG is a high powered hard working committee where every member brings significant knowledge and intellect and a passion to contribute to the co-design of the Alliance’s policies and procedures, genomic information and communication. It is very collaborative, consultative and there is a high level of warmth, trust and respect amongst members for each other. The CAG reinforces the aim of patient centred care and partnering with consumers.

The CAG won the ‘Outstanding Achievement by a Volunteer – Better Care Victoria Innovation Award’ in the 2017 Minister for Health Volunteer Awards, in recognition of the group's innovation in bringing community views and advice to the implementation of genomics in health care [33].

## Discussion

Widespread recognition now exists that patient involvement has the potential to improve the quality of decision-making and increase fairness, responsiveness and legitimacy of programs of work. Each initiative should be tailor-made in terms of whom to involve, how to involve them and how to value their contributions [34]. The strength of the CAG was the relationship it developed with the Program Management Team. This has been identified as a key factor in successful community engagement and involvement [35]. A literature review on relationship building for community-academic collaborations in health research and innovation was conducted in 2018 [36], finding that such relationships assist in better matching programs of work with societal values, needs and expectations. The gold standard requires open, transparent, trustful and ongoing relationships between community partners and researchers. There is a need for common goals – and to ensure that those needs and goals are made explicit. Common commitment to project goals is also important. Researchers need to explain their reasons for wanting to form a relationship; negotiate formal roles and expectations; disclose and share information; and keep community members informed about the findings. The partnership met these goals to varying degrees. This resonates with the analysis of effective participation in health policy and planning where ‘political’ commitment, partnership synergy, inclusiveness and deliberativeness are important [3]. Partnership synergy was defined as ‘the ability to work together by combining resources in order to produce an output that cannot otherwise be achieved by single agents’ [3]. Such synergy requires a quality working relationship, trust and a degree of shared identity in searching for a group solution. ‘Deliberativeness’ refers to the quality of the discussion on issues under question and the quality of the information provided [3]. The CAG was invariably provided with clear presentations and materials to inform discussions, contributing to our willingness to work, to listen and provide our input.

Personal qualities and competence are an important part of relationship building, from the beginning to the end of any collaborative partnership. Within the Alliance goodwill was shown to community partners, for example in running the workshop for patient groups, and through the many interactions over the course of the program including informal contacts. The efforts of the CAG were also rewarded as evidenced by their successful nomination for a Ministerial award, and through assisting with preparation of posters and presentations. This led to empowerment of members of the CAG and the ability to communicate informatively, aspects that Stallings [37] identified as being important. The CAG provided a constant reminder to the Project team of patient values and their importance in decision making [38]. Medical research should benefit society at large. Involving the community may not only increase the quality, but can also push research towards generating greater societal benefits and relevant outcomes for the community [39]. Health care planning and having to make specific decisions through being presented with available alternatives, may prompt patients to consider or reconsider stated values, particularly as circumstances change. Involvement of family members and cultural norms are important factors [36], particularly in the area of genomics where values other than in addressing an individual’s own needs may be factored in when making decisions.

A partnership approach with trusted relationships can accomplish a shared understanding of public involvement in research among different stakeholders and embed a sustainable and meaningful implementation of public involvement activities [39]. The CAG Activity register highlighted the diversity of CAG contributions to the Melbourne Genomics program. In the earlier stages of the program, where an objective of the program was to find answers and possible solutions for patients, they contributed to resources and tools for participants. Later in the program they partnered with the Project Management Team in outward looking activities such as the workshop for patient advocates, interacting with external experts and presentations at symposia.

In large complex clinical studies, in which multiple partner organizations are involved, we tend to forget that our joint effort is not just scientific research or making a new test available, it is finding actual medical solutions for patients. The work of the CAG provided a constant reminder of this goal. Community involvement may not always be easy and needs time to grow. But as the feedback from the Alliance team illustrates, it is an enriching experience for community members, the Program Management Team, clinicians and researchers. Benefits were broader than the scope of the program. Today, there is uniform appreciation for the important contributions of the CAG in ensuring a patient-care design across the program, with this model being adopted by other collaborations in genomics around Australia. The group is helping to build an aware and engaged community, which will be essential to genomic medicine becoming part of everyday health care.

### Strengths and enablers of the Alliance CAG (Table 3)

The strong governance structure of the Alliance provided a firm foundation for the CAG and supported it in its work. Strong organisational support was provided, with one Program Team member responsible for the administration of the CAG and in preparing meetings and consultations.

Active attendance at CAG meetings by the Executive Director and Team Leader of the Program Management Team meant that executive level support was evident, and that the CAG was kept informed. The collaborative spirit of the Program Team was also evident at meetings and in presentations given by Flagship representatives. Of equal importance was the commitment given by experienced CAG members to the Alliance, and their links to their communities, resulting in better research and health care.

Consumer and community engagement in research is increasingly valued in a contemporary health care environment that seeks to genuinely partner with consumers and the wider community.

There was clearly a commitment to patient involvement from the key research leads from the outset of the study, but with less knowledge of what good patient involvement looks like. Establishing such a group and fully integrating it with the activities across the Alliance required proactive effort.

Having senior management and executives recognise and advocate for the importance of patient involvement from the start of the program fostered a sense of empowerment and commitment among CAG members and ensured sustainability of the community engagement.

Allowing time to plan was valuable, where the CAG and its individual members elected where they wanted to be involved and how. There was also a flexible approach to interactions, with face-to-face meetings as a group and in smaller groups, telephone and internet calls, and tasks undertaken by e-mail. At a structural level, resources were allocated in the form of honoraria and expenses paid and a CAG co-ordinator position was established to show organisational commitment (see [9]).

### Limitations

Some CAG members had no formal training in genomics and the scientific aspects relevant to the Alliance program to prepare them for their role. The first CAG Chair spent a great deal of time answering questions of CAG members on clinical utility of genomics and related topics. Members were able to attend Alliance events including lectures and were on working groups that helped increase their knowledge through background reading. It was also difficult for a new member joining the CAG after the Demonstration phase.

Lack of diversity among consumer representatives—including from Aboriginal and Torres Strait Island communities—will have to be addressed as the Alliance moves forward in its work [40]. As identified by Anderst [9], this is a general problem in Australia despite its diverse population. CAG members were able to connect the Program Team to diverse community groups that they had connections to, and alert the team to the need to consult with diverse communities through their leaders.

The CAG monitored its own activities through the Activity registry. No formal evaluation of the CAG was undertaken. Monitoring and evaluation with locally relevant questions is important to ensure meaningful and collaborative engagement, as demonstrated in Canada [41]. Evaluation was paramount in other aspects of the program where people across the Alliance seemed more willing to reach agreement on decisions where the outcome—a policy, guideline or software—would be subject to evaluation, with the possibility of positive adaptations and changes in approaches in the future [21]. Could this same principle also apply to community engagement? As Han 2021 has highlighted [11], this is an evolving area for community engagement. As part of the final phase of the Alliance work a formal evaluation of the CAG is planned.

The CAG was an advisory group to the Alliance Management Team with limited ability to influence flagships once undertaking their areas of incorporating genomics into clinical services, although the CAG may have acted as a role model for them within their health services. Greater communication pathways within the Alliance [21] may have overcome this limitation.

## Conclusions

The CAG added value to the clinical service-research genomic program of work, right from the beginning through to completion of the program. The balanced trusting relationship that developed between the CAG, the Program Team and its governance structure has been of great value and a significant achievement for the Alliance. CAG input into project deliverables, particularly in the Demonstration phase, was very tangible. Their less tangible contributions to the project were also important. Contributions included presentations at annual meetings, direct interactions at those meetings with the Alliance members, and interactions with visitors and external experts. Taking part in consultations with external experts and with representatives from state and federal governments may also have influenced mindsets. The model of involvement worked well for our research-to-clinical service genomics program of work.

The CAG activities followed the full spectrum of information sharing and consultation through to co-design and partnership. The CAG made multifaceted contributions to the work of the Alliance by being involved at several levels of participation and in all tiers of activity including governance, development of policy and procedures, planning and evaluation. The CAG members became an accepted part of the Alliance, by its leadership and Program Team through their contributions both as a group and as individuals. The CAG members drew on their contacts in their communities and applied their skills and knowledge to many aspects of the work. With leadership commitment, working relationships have been built and a level of trust instilled [42] to work toward ensuring a patient-care model of delivery of genomics.

## Data Availability

The data that support the findings in this study are available from the corresponding author upon reasonable request.

## Abbreviations

CAG: Melbourne Genomics Health Alliance Community Advisory Group
CHF: Consumers Health Forum of Australia
HTA: Health Technology Assessment
The Alliance: Melbourne Genomics Health Alliance

## Declarations

### Authors' information

This paper was initiated by the lead author, who collated meeting minutes, contents of the ‘Activities register’, CAG presentations and Alliance reports. A poster presentation was given at the Consumers Health Forum (CHF) first Australian and New Zealand Summit on consumer leadership and experience in health care (18–19 March 2021) (https://chf.org.au/events/chf-summit-2021-shifting-gears). The poster had input from all the authors of this case report and is the basis of the present paper. Members of the Project Management Team made valuable contributions to the preparation of this manuscript.
